# Changes in the Amino Acid Composition of Bogue (*Boops* *boops*) Fish during Storage at Different Temperatures by ^1^H-NMR Spectroscopy

**DOI:** 10.3390/nu4060542

**Published:** 2012-06-20

**Authors:** Alessandra Ciampa, Gianfranco Picone, Luca Laghi, Homa Nikzad, Francesco Capozzi

**Affiliations:** 1 Department of Food Science, University of Bologna, Piazza Goidanich 60, Cesena 47023 (FC), Italy; Email: alessandra.ciampa2@unibo.it (A.C.); l.laghi@unibo.it (L.L.); 2 Interdepartmental Centre for Industrial Agri-Food Research c/o Campus of Food Science, University of Bologna, Piazza Goidanich 60, Cesena 47521 (FC), Italy; Email: gianfranco.picone2@unibo.it; 3 Department of Veterinary Medical Science, Alma Mater Studiorum–University of Bologna, Via Tolara di Sopra 50, 40064 Ozzano dell’Emilia (BO), Italy; Email: homa.nik@alice.it; 4 Center of Magnetic Resonance, University of Florence, Via L. Sacconi 6, Sesto Fiorentino 50019 (FI), Italy

**Keywords:** fish, freshness index, ^1^H-NMR, free amino acids (FAAs), quality assessment, metabolic profile, storage

## Abstract

Nuclear magnetic resonance spectroscopy was employed to obtain information about the changes occurring in Bogue (*Boops boops*) fish during storage. For this purpose, ^1^H-NMR spectra were recorded at 600 MHz on trichloroacetic acid extracts of fish flesh stored over a 15 days period both at 4 °C and on ice. Such spectra allowed the identification and quantification of amino acids, together with the main organic acids and alcohols. The concentration of acidic and basic free amino acids was generally found to increase and decrease during storage, respectively. These concentration changes were slow during the first days, as a consequence of protein autolysis, and at higher rates afterward, resulting from microbial development. Two of the amino acids that showed the greatest concentration change were alanine and glycine, known to have a key role in determining the individual taste of different fish species. The concentration of serine decreased during storage, as highlighted in the literature for frozen fish samples. Differences in the amino acids concentration trends were found to be related to the different storage temperatures from day 4 onwards.

## 1. Introduction

The amino acid composition of fish muscle proteins has been known for a long time to be remarkably constant across different species of fish [1]. Fish muscles contain from 1 to 5 g of free amino acids for every 100 g of protein, characterized by high quantities of taurine, histidine, glutamic acid, alanine, aspartic acid, leucine and lysine and by lower quantities of cysteine, tryptophan, methionine and tyrosine [2]. The pattern of free amino acid concentrations, rather than the composition of the amino acids bound to the proteins, is known to depend on the fish species. One of the reasons is that some free amino acids act as cell osmoregulators, so that their relative amount with respect to other free amino acids that are not involved in cellular osmotic regulation, can be modulated by the salt concentration in the fish habitat [1].

Concentration and pattern of free amino acids are also very sensitive to the changes occurring in fish muscle during storage. During the first hours following death such characteristics are modulated by autolysis, the degradation of muscle constituents by endogenous enzymes. This degradation is known to be pH, temperature and fish processing dependent [3].

The pH, decreasing upon death due to the transformation of glycogen into lactate, modulates the exit of the lytic enzymes from lysosomes. In this respect, it is of importance to note that catching methods that involve intense fish struggling lead to a greater transformation of glycogen into lactic acid, thus to a more powerful autolysis. The rates of these reactions are generally directly related to temperature, with an optimum around 36 °C for most of the lytic enzymes. Among the processing treatments able to modulate autolysis, evisceration may be of primary importance, as some proteolytic enzymes are known to be located in the gut [4].

The greatest modifications to the amount and pattern of free amino acids occur when the conditions are favorable for bacterial development. This phenomenon has contrasting effects on free amino acid concentrations as these molecules are extracted from proteins by lytic reactions and, in parallel, transformed into secondary products, some of which lead to malodors [5].

Autolysis and bacterial spoilage are responsible for changes occurring in the concentrations of adenosine-5′-triphosphate (ATP), adenosine-5′-diphosphate (ADP), adenosine-5′-monophosphate (AMP), and inosine-5′-monophosphate (IMP), which are quantitatively converted to inosine (HxR) and hypoxanthine (Hx) [6,7]. For this reason, all such molecules are often quantified to evaluate the quality loss during fish storage, and their relative amounts are combined together in a score to express fish freshness. One of the first conventional quality indices setup to take the degradation of such nucleotides into consideration is the K index [8], defined as

                  K (%) = ([HxR] + [Hx])/([ATP] + [ADP] + [AMP] + [IMP] + [HxR] + [Hx])

As free amino acids are key molecules in both autolysis and biological spoilage reactions, their observation may offer alternatives to the K index and its variants to follow fish quality loss during storage. Proton nuclear magnetic resonance (^1^H-NMR) can be in turn a suitable technique for this purpose, as suggested by recent papers published by some of the authors of the present work [9]. 

Picone *et al*. [10] observed free amino acids and other low molecular weight molecules using ^1^H-NMR to discriminate farmed specimens of Gilthead Seabream (*Sparus aurata*) according to the aquaculture system employed. Savorani *et al*. [11] employed NMR spectra to find out discriminant NMR intervals’ spectra through *i*ECVA [11,12], whilst Capozzi *et al*. [13] outlined a new algorithm that was able to correct the dilution errors during sample preparation affecting the quantification of serine, valine, histidine, phenylalanine and other amino acids.

Prompted by these encouraging results, we decided to apply ^1^H-NMR spectroscopy to monitor the changes occurring in the free amino acid pool during fish storage, to investigate the possibility of using such changes as storage quality indices. The fish chosen for the investigation belong to the Bogue (*Boops boops*) species, whose amino acidic composition is still uncharacterized in the literature, even if this species is commercially important and very popular in several Mediterranean countries [14,15].

## 2. Experimental Section

### 2.1. Sampling

All samples of Bogue fish, provided by Magna Grecia Mare—Portus Veneris (Leuca, Lecce, Italy), were caught in spring and brought to the laboratory in about ten hours, by means of polystyrene boxes filled with ice flakes. Ungutted fishes were divided into two groups. The first was stored at 4 °C and samples taken 0, 2, 4, 6, 8, 10, 11 days after catching. The second group was stored on ice and sampled 0, 2, 4, 6, 8, 10, 15 days after catching. The fishes were prepared for further processing by slicing them in a room at 4 °C and storing the flesh at −80 °C. 

### 2.2. Sample Preparation for ^1^H-NMR Analysis

At each sampling time and temperature a trichloroacetic acid extraction (TCA) was performed on three fish samples, by following the procedure set up by Boland *et al* [16]. For this purpose, 25 g of fish muscle was added to 50 mL of 7.5% (w/w) TCA and minced by means of a vertical homogenizer (Ultra-Turrax, Ika^®^). The resulting product was filtered with filter paper (No. 4) from Whatman (Little Chalfont, Buckinghamshire, HP7 9NA, UK). The pH of a 1 mL aliquot was adjusted to 7.8 using 9 M KOH in an Eppendorf microfuge tube and centrifuged at 14 K rpm for 5 min in order to remove potassium trichloroacetate precipitate. The so obtained supernatant was stored at −80 °C until ^1^H-NMR measurements were performed.

### 2.3. ^1^H-NMR Measurements

The samples were prepared for NMR analysis by adding 160 µL of a D_2_O solution of 3-(trimethylsilyl)-propionic-2,2,3,3-d4 acid sodium salt (TSP) 6.25 mM to the thawed samples.

^1^H-NMR spectra were recorded at 298 K with a Bruker (Milano, Italy) AVANCE spectrometer operating at a frequency of 600.13 MHz, equipped with an autosampler with 60 holders.

Each spectrum was acquired using 32 K data points over a 7211.54 Hz spectral width and adding 256 transients. A recycle delay of 5 s and a 90° pulse of 11.4 μs were set up. Acquisition time (2.27 s) and recycle delay were adjusted to be 5 times longer than the T_1_ of the protons under investigation, which has been considered to be not longer than 1.4 s. Saturation of residual water signal was achieved by irradiating it during the recycle delay at δ equal to 4.703 ppm. Each spectrum was processed with MestReC 4.9.8.0 (Mestreab Research SL, Spain) by manually adjusting phase and base-line and applying a line broadening factor of 0.5 Hz.

The peaks were assigned by comparing their chemical shift and multiplicity with the literature [11]. When peaks due to different protons of the same molecule were identified, both were employed for the quantification.

## 3. Results and Discussion

**Figure 1:**
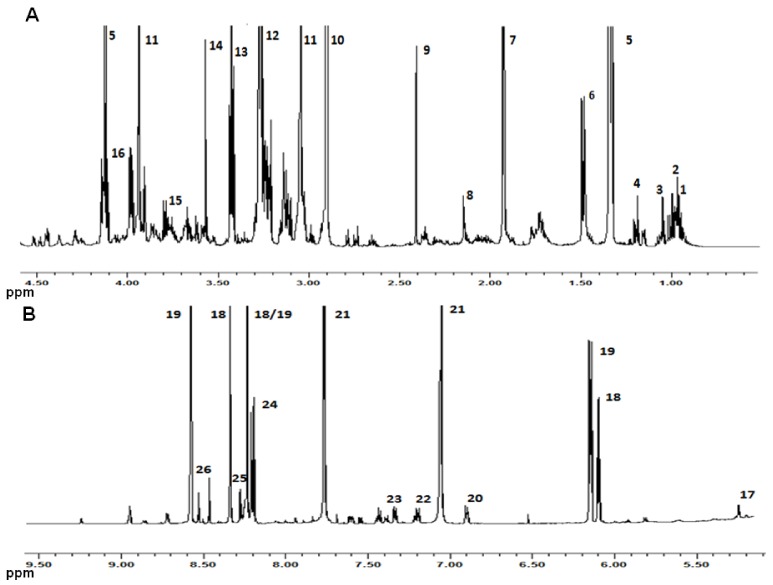
600.13 MHz ^1^H NMR spectrum of TCA Bogue fish extract. The numbers refer to the assignments reported in Table 1.

A typical ^1^H-NMR spectrum obtained during the present investigation is shown in Figure 1. Three groups of peaks could be identified. The peaks with the highest intensity, accounting for 30% of the total spectra area, pertain to trimethylamine-*N*-oxide (TMAO), trimethylamine (TMA) and creatine and phosphocreatine. In the region between 8.16 and 8.60 ppm the peaks employed to calculate the K index could be identified. The remaining peaks could be mainly ascribed to amino acids and, to a minor extent, to organic acids and short chain fatty acids. At the chemical shifts of the amino acids sharp peaks could be identified, with a width at half height around 1 Hz, which could be assigned to amino acids in the free form or pertaining to low molecular weight peptides. At the base of some of such peaks, macromolecules and aggregates gave rise to much broader signals, which could not be discriminated from the baseline noise. Such different behavior is caused by the inverse relationship existing between signal linewidth and (i) the nuclear relaxation rate, being shorter for slow tumbling macromolecules; and (ii) molecular anisotropy, being longer for denatured and disordered proteins [17].

Table 1 lists the NMR signals of the amino acids involved in the fish spoilage during the present investigation. The resonances used in the integration were chosen from among those belonging to the corresponding signal multiplet which was found to be only marginally overlapping with other signals. Nevertheless, for some amino acids a clean signal was not found, and for this reason their variation was given in a cumulative way (e.g., Ile, Leu and Val) or not given at all (e.g., Lys and Pro). In addition, the table lists the assignments of other substances, whose concentration could help in understanding which phenomenon, autolysis or bacteria development, was mainly responsible for the identified fluctuations of the amino acid concentrations.

**Table 1:** Summary of the metabolites identified in the 600.13 MHz ^1^H NMR spectrum of the aqueous extract of Bogue fish.

Compound	Assignment	^1^H (ppm)	Multiplicity
Isoleucine (Ile) ^1^	δ-CH_3_	0.94	t
Leucine (Leu) ^2^	δ′-CH_3_	0.96	d
Valine (Val) ^3^	γ-CH_3_-γ′-CH_3_	1.00–1.05	dd
Ethanol ^4^	CH_3_	1.19	t
Lactate (La) ^5^	β-CH_3_	1.33	d
Alanine (Ala) ^6^	β-CH_3_	1.49	d
Acetate ^7^	CH_3_	1.93	s
Methionine (Met) ^8^	S–CH_3_	2.14	s
Succinate ^9^	α,β-CH_2_	2.41	s
Trimethylamine (*N*-TMA) ^10^	N–CH_3_	2.90	s
Creatine/Phosphocreatine ^11^	N–CH_3_ and N=C	3.04	s
Oxide Trimethylamine (*N*-TMAO) ^12^	N–CH_3_	3.27	S
Taurin (Tau) ^13^	N–CH_2_	3.42	t
Glycine (Gly) ^14^	α-CH	3.56	s
Glutamate (Glu) ^15^	α-CH	3.75	t
Creatine/Phosphocreatine ^11^	N–CH_2_	3.94	s
Serine (Ser) ^16^	β-CH	3.98	dd
α-Glucose (α-GLC) ^17^	CH-1	5.24	d
Inosine (HxR) ^18^	CH-1′, ribose	6.10	d
Inosine 5′-monophosphate (IMP) ^19^	CH-1′, ribose	6.14	d
Tyrosine (Tyr) ^20^	C_3,5_H, ring	6.88	d
Histidine (His) ^21^	C_2_H ring/C_4_H ring	7.06/7.77	s
Tryptophan (Trp) ^22^	C_5_H ring	7.19	t
Phenylalanine (Phe) ^23^	CH-2,6	7.32	m
Hypoxanthine (Hx) ^24^	CH-8	8.19	s
Hypoxanthine (Hx) ^24^	CH-2	8.21	s
Inosine (HxR) ^18^	CH-8	8.233	s
Inosine 5′-monophosphate (IMP) ^19^	CH-8	8.236	s
Adenosine 5′-triphosphate (ATP) ^25^	CH-8	8.27	s
Adenosine 5′-diphosphate (ADP) ^25^			
Adenosine 5′-monophosphate (AMP) ^25^			
Inosine (HxR) ^18^	CH_2_, ring	8.33	s
Formate (Fo) ^26^	CH	8.46	s
Inosine 5′-monophosphate (IMP) ^19^	CH_2_, ring	8.57	s

When the content evolution of a mixture has to be evaluated by comparing different spectra registered from different samples, the spectra cannot be directly compared, even when acquired with the same parameters as in the present case. Several factors are in fact known to alter the sensitivity of the instrument from sample to sample, leading to variations in the relationship between concentration of the analytes and the area of the corresponding peaks [18].

To avoid this source of error when measuring content evolution of a mixture, most often a referring standard is selected whose concentration is constant among the different samples. The spectra intensity is then scaled to this concentration, a procedure called “normalization” or vertical scaling [13]. 

When dealing with biological samples, it is common practice to employ the total area of the spectrum as a referring standard, assuming that all the substances interconvert to each other during storage, leading to an almost constant total area [19]. This option could not be utilized for the samples analyzed during the present investigation, as protein hydrolysis occurring during conservation led to a progressive increase of the spectral total area. The same phenomenon potentially influences the concentration of every molecule characterizing the samples, so that none of them could be confidently employed as an endogenous internal standard. Another possibility with biological samples consists in adjusting the spectral vertical scale to the area of an added molecule, the so-called added internal reference standard. When the investigated samples are liquids extracted from solids, the standard addition can only be in the last step of the sample preparation process. The relative area of the internal standard becomes thus sensitive to the variability induced by the extraction efficiency, which in turn is variable among samples undergoing time dependent structural degradation [20]. In addition, an added molecule may represent an unreliable concentration reference if interactions with macromolecules characterizing the sample occur [21].

An alternative to all the above mentioned normalization procedures can be offered by the presence of a small pool of metabolites known to be present in similar concentrations in every sample and known to belong to a closed inter-conversion pathway, so that their total molar amount can be considered as constant during storage. This is the case of the ATP degradation pathway, which in *post mortem* conditions is ultimately converted to hypoxanthine, via the by-products included in the calculation of the K-index [6]. To test the possibility of scaling towards the area of the peaks due to ATP and byproducts, a procedure we may call K-index normalization (KIN), the total area of these molecules was calculated on the raw spectra before normalization. In spite of the possible sources of error potentially affecting the spectra, the samples analyzed at the same storage times gave an average RSD of 3.3% and no statistically significant differences were found among the time points. The KIN method was thus elected as the normalization procedure for the present work.

**Figure 2:**
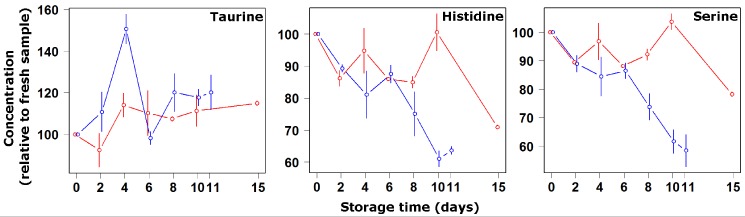
Concentration changes relative to fresh samples of taurine, histidine and serine during storage at 4 °C (blue symbols and lines) and on ice (red symbols and lines).

**Figure 3:**
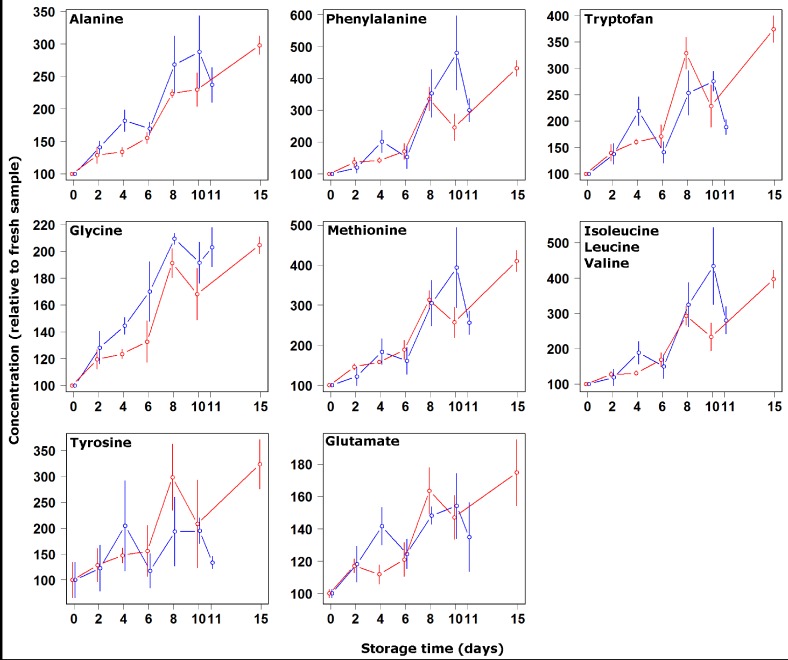
Concentration changes relative to fresh samples of alanine, phenylalanine, tryptophan, glycine, methionine, isoleucine-leucine-valine, tyrosine and glutamate during storage at 4 °C (blue symbols and lines) and on ice (red symbols and lines).

Figure 2 and Figure 3 present the fluctuation during storage on ice and at 4 °C of the molecules followed during the present investigation. The amino acids could be divided into three groups according to the trend of their concentration. The first group was represented by taurine only, whose concentration did not significantly change during storage. This was not unexpected, since this amino acid is not employed by organisms as a protein constituent, thus it is not involved in the lytic processes going on during autolysis reactions or bacterial development. Indeed, results in accordance with this observation can be found in the literature for several kinds of fishes and fish preparations, stored from −20 °C [22] to 25 °C [23]. 

The second and third groups comprised the amino acids whose concentration increased and decreased, respectively, as a consequence of storage at 4 °C. The former group was composed of alanine, phenylalanine, tryptophan, glycine, methionine, isoleucine-leucine-valine, tyrosine and glutamate, while the latter group was made up of histidine and serine only. The decrease in concentration of the basic amino acids shows that their solubilization from muscle proteins was slower than their transformation into byproducts, namely biogenic amines through decarboxylation. Such finding, together with the parallel increase of the acidic amino acids, is well documented at ambient temperature for a variety of fishes and transformed products based on them [24,25]. Kiesvaara [26] set up specific quality indices for salted herrings based on such knowledge. At room temperature, moreover, there is a general agreement about the decrease of methionine during storage [27]. The data collected for the present investigation seems to confirm the findings. Exceptions are represented by serine, which is acidic but decreases, and methionine, which increased in concentration at both investigated temperatures. Such trends appear to be more similar to those observed in the literature for frozen samples. As an example, Jiang *et al*. [22] found decreasing concentrations of serine in frozen samples of mackerel, amberfish, mullet and carp during storage, and at the same time an increasing concentration of methionine. The fact that at both temperatures the tested methionine did not decrease is interesting from a consumer perspective, as the catabolism of this amino acid is known to be primarily responsible for methylmercaptan and dimethylsulphide formation, molecules strongly related to development of off-flavors [28]. 

By focusing on the relationship between amino acid concentrations and storage time, it was possible to divide the observed molecules into two categories. The concentration of alanine, tryptophan, glycine methionine and tyrosine were observed to change during storage with a constant trend. In contrast, the other molecules seemed to be characterized by a slow change until day 4 and by a higher change rate afterwards. In this respect it must be noted that the concentration of some of the latter molecules, in particular histidine and serine, changed at similar rates in the two storage methods until day 4, and at markedly different rates afterwards. Such a two-phase observation could be rationalized considering that the enzymes leading to autolysis are known to be poorly influenced by temperature, being still active even at temperatures as low as −17 °C [3]. The slow rate change until day 4 can thus be considered mainly due to autolysis, the fast rate change occurring afterwards due to bacterial development. Indeed, the concentration of some of the mentioned molecules seem to reproduce a bacterial development curve [5] characterized by: (i) a lag phase in which the Bogue can be considered as fresh. During this phase the concentration of some amino acids undergoes fluctuations appreciated by the consumer in specific cases. Free glycine, for example, is known to be important for the individual taste of different fish species [29]; (ii) an exponential multiplication phase, typically characterized by development of off-flavors; and (iii) a stationary phase during which the concentration of some free amino acids start to decrease.

**Figure 4:**
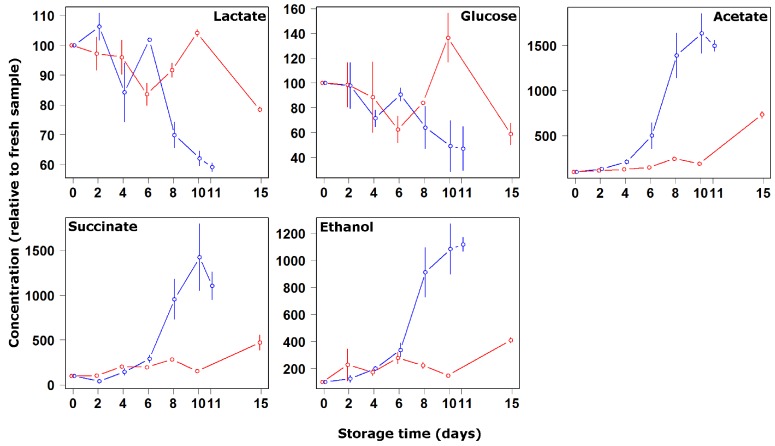
Concentration changes relative to fresh samples of lactate, glucose, acetate, succinate and ethanol during storage at 4 °C (blue symbols and lines) and on ice (red symbols and lines).

To confirm the findings based on the quantification of the free amino acids, other molecules involved in the microorganisms’ development were quantified (Figure 4). Glucose and lactate are recognized as a substrate for most of the microorganisms involved in the spoilage of fish, glucose being consumed at first instance, followed by lactate and amino acids [30]. At the same time, acetate, succinate and ethanol typically accumulate in the medium, as a consequence of such development. Indeed, during the present investigation a 40% decrease of both glucose and lactate was noticed for the samples storage at 4 °C, whilst acetate, succinate and ethanol increased from the fourth day in samples stored at 4 °C and after 15 days in the samples stored on ice. 

## 4. Conclusions

In the present work, changes of Bogue fish muscle composition were followed as a consequence of storage at 4 °C and on ice. For this purpose, ^1^H-NMR spectra were recorded on Bogue fish muscle TCA extracts and then normalized to the total area of the peaks pertaining to ATP and its degradation products. Through such amplitude adjustment, preferred to other kinds of vertical scaling procedures described in the literature, the effects of storage on the concentration of 13 amino acids in the free form could be registered. The concentration of some of them was observed to increase in the fish flesh as a consequence of enzymatic reactions, during the first days of storage, and due to bacterial development afterwards. Histidine concentration was observed to decrease, as a consequence of decarboxylation leading to histamine. The storage temperature seemed to mainly affect bacterial development rate, modulating the amino acid concentrations starting from day 4 of storage. The amino acids profile was shown to be sensitive to the phenomena leading to the compositional changes occurring during fish storage. Its evaluation, through ^1^H-NMR spectroscopy or other less expensive techniques, appears to be a promising source of parameters suitable for the assessment of freshness, as an alternative to the K-index or analogue indices. It is important to stress here that NMR spectroscopy must be considered as a “non targeted” technique able to quantify and evaluate kinetics for unselected compounds, even *a posteriori*, e.g., after a pool of spectra is recorded on a population of samples and the multivariate analysis points out some interesting features in the molecular profile, that may be suitable for the development of new parameters related to quality and freshness.
